# Emergence and Phylodynamics of *Citrus tristeza virus* in Sicily, Italy

**DOI:** 10.1371/journal.pone.0066700

**Published:** 2013-06-20

**Authors:** Salvatore Davino, Anouk Willemsen, Stefano Panno, Mario Davino, Antonino Catara, Santiago F. Elena, Luis Rubio

**Affiliations:** 1 University of Palermo, Palermo, Italy; 2 IEMEST, Palermo, Italy; 3 IBMCP, CSIC-UPV, Valencia, Spain; 4 University of Catania, Catania, Italy; 5 Parco Scientifico e Tecnologico della Sicilia, Cataia, Italy; 6 The Santa Fe Institute, Santa Fe, New Mexico, United States of America; 7 IVIA, Moncada, Valencia, Spain; Duke-NUS Gradute Medical School, Singapore

## Abstract

*Citrus tristeza virus* (CTV) outbreaks were detected in Sicily island, Italy for the first time in 2002. To gain insight into the evolutionary forces driving the emergence and phylogeography of these CTV populations, we determined and analyzed the nucleotide sequences of the p20 gene from 108 CTV isolates collected from 2002 to 2009. Bayesian phylogenetic analysis revealed that mild and severe CTV isolates belonging to five different clades (lineages) were introduced in Sicily in 2002. Phylogeographic analysis showed that four lineages co-circulated in the main citrus growing area located in Eastern Sicily. However, only one lineage (composed of mild isolates) spread to distant areas of Sicily and was detected after 2007. No correlation was found between genetic variation and citrus host, indicating that citrus cultivars did not exert differential selective pressures on the virus. The genetic variation of CTV was not structured according to geographical location or sampling time, likely due to the multiple introduction events and a complex migration pattern with intense co- and re-circulation of different lineages in the same area. The phylogenetic structure, statistical tests of neutrality and comparison of synonymous and nonsynonymous substitution rates suggest that weak negative selection and genetic drift following a rapid expansion may be the main causes of the CTV variability observed today in Sicily. Nonetheless, three adjacent amino acids at the p20 N-terminal region were found to be under positive selection, likely resulting from adaptation events.

## Introduction

Viruses, in particular those with RNA genomes, are the most abundant parasites infecting animals, plants, and bacteria. They have a high socio-economic impact on welfare of humans and on productivity of livestock and agriculture. RNA viruses also have a great potential for rapid evolution due to the high mutation rates, large population sizes and short generation times [1]. This rapid evolution means that epidemiological and evolutionary processes occur on a similar time scale of a few years and that they may interact conditioning the spatiotemporal incidence and phylogenetic patterns. Phylodynamics, the synthesis between epidemiology and evolutionary biology, can provide relevant information to understand the evolution of virulence, the emergence of new viral diseases and to design more efficient strategies for disease control [2], [3]. Many studies on the phylogeography or phylodynamics of human and animal viruses on different geographical scales have been performed [4]–[9] but these studies are still scarce for plant viruses and are mostly restricted to viruses infecting annual crops [10]–[13]. Epidemiology and evolution of plant viruses infecting perennial hosts may differ from those of plant viruses infecting annual crops, in which the host is replaced each year, and from those of animal/human viruses that are mobile hosts. Also, to our knowledge, phylodynamics associated with the colonization of a new geographical area by a plant virus has not been addressed. Here, we studied the colonization of citrus growing areas of Sicily, Italy by *Citrus tristeza virus* (CTV; genus *Closterovirus*, family *Closteroviridae*) and evaluated the temporal and spatial patterns of CTV spread, the potential effect of different host species, and the evolution of CTV isolates differing in virulence.

CTV has long flexuous virions consisting of two coat proteins, the major (CP), covering most of the genomic RNA, and the minor (CPm) located to one of the virion ends [14]. CTV genome is a positive-sense, single-stranced RNA of 19.3 kb with 12 open reading frames (ORFs) and two untranslated regions (UTRs) of about 107 and 273 nt at its 5′ and 3′end, respectively. ORFs 1a and 1b are directly translated from the genomic RNA and encode proteins involved in RNA replication. The other ORFs are expressed via 3′-coterminal subgenomic RNAs and encode proteins p6, p65, p61, p27, p25, p18, p13, p20, and p23, required for virion assembly and cell-to-cell movement (p6, p65, p61, p27, and p25), asymmetrical accumulation of positive and negative strands during RNA replication (p23), suppression of post-transcriptional gene silencing (p25, p20 and p23), invasion of some host species (p33, p18 and p13), or superinfection exclusion between genetically related CTV isolates (p33) [15]–[17].

CTV is the causal agent of some of the most economical important diseases in citrus worldwide [15]. This virus has a narrow natural host range essentially restricted to some species of the genera *Citrus* and *Fortunella* in the family *Rutaceae* and infects only phloem-associated cells. Depending on virus strains and on host species or scion–rootstock combination, CTV may cause three distinct syndromes [15], [18]: (i) tristeza, a decline syndrome affecting citrus species grafted on sour orange or lemon rootstocks; (ii) stem-pitting, stunting, reduced yield and low fruit quality regardless of the rootstock used; and (iii) seedling yellows, characterized by stunting, small yellow leaves, reduced root system and sometimes a complete cessation of growth of sour orange, grapefruit or lemon seedlings.

CTV has been disseminated to almost all citrus-growing countries through the infected budwood propagation and subsequent local spread by aphid vectors [15]. The most destructive epidemics occurred in Argentina (1930), Brazil (1937), California, USA (1939), Florida, USA (1951), Spain (1957), Israel (1970), and Venezuela (1980); but important outbreaks have also been reported from Cyprus (1989), Cuba (1992), México (1995), Dominican Republic (1996), and, more recently, in Italy (2002). Here, two foci of mild CTV isolates were identified in Apulia (Southeastern part of the Italian peninsula) and in Cassibile (Eastern part of Sicily), and a third focus of severe CTV isolates in Belpasso, also in Eastern Sicily about 80 Km away from Cassibile [19]. Severe CTV isolates induce seedling yellows in sour orange and vein corking in Mexican lime, whereas mild CTV isolates are symptomless in sour orange and produce only a slight vein clearing in Mexican lime.

Genetic and evolutionary studies on CTV have revealed important features such as conservation of genomes in distant geographical regions with slow evolutionary rates [20]–[22]; uneven distribution of variation along the genome [23], [24]; and frequent recombination between divergent genomic variants [21], [25], [26]. Population genetics studies showed that intense gene flow and negative selection shaped the genetic structure of the long-established CTV populations in California and Spain [21], [27]. However, a complete understanding of the dynamics of CTV evolution and epidemiology in spatial and temporal scales remains an important goal. Also, the emergence and the evolutionary processes of CTV in new colonized areas have never been examined. In this regard, recent CTV outbreaks in Sicily after introduction of mild and severe genetically distinct isolates in two nearby foci offered an opportunity to analyze the emergence and dynamics of CTV colonization.

In this study, we report the results from an exhaustive CTV survey carried out in all citrus-growing areas of Sicily since the first outbreaks in 2002 until 2009 and the analysis of the p20 gene (549 nt) nucleotide sequences of 108 representative CTV isolates. The spatial and temporal genetic variation of CTV in Sicily was investigated using a phylodynamic-based approach to gain insight in the processes involved in the emergence, spatial-temporal spread and evolutionary dynamics of CTV.

## Results

### Spatio-temporal Prevalence of CTV in Sicily

**Figure 1:**
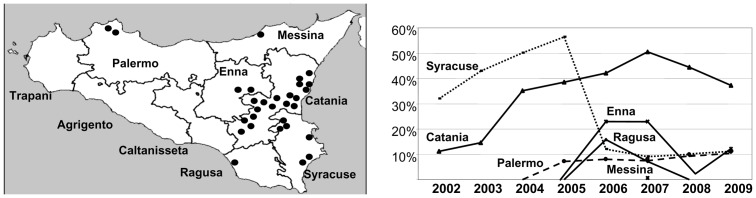
Incidence (percentage of CTV-infected citrus trees) per year in each of the eight Sicilian provinces from 2002 to 2009.

Samples were collected randomly from the main citrus areas of different Sicilian provinces since 2002, when the first outbreaks of CTV occurred, until 2009. The analyses of samples from 67,922 citrus trees revealed that about half of them were infected by CTV (Table S1 in Tables S1). Most were concentrated in an intensive citrus-growing region of about 3000 km^2^ around the first outbreak foci detected [19] which included parts of the Catania, Syracuse and Enna provinces (Fig. 1). The prevalence of CTV increased from 2002 reaching a maximum peak of about 50% in Syracuse in 2005 and in Catania in 2007, followed by a moderate decrease in Catania until 37.4% whereas in Syracuse plummeted to about 10% (Fig. 1). In Enna, CTV was found in 2006 and 2007 with a prevalence of about 20% and in 2009 with a prevalence of about 10%. In the Northwest, CTV was detected in Palermo in 2005 with a steady prevalence of about 10% and sporadically in the Northeast, Messina, in 2007. In the South, CTV was only found in Ragusa in 2006 and 2007 with a prevalence around 10% whereas it was never detected in the western provinces of Trapani, Agrigento and Caltanissetta.

### Phylogenetic Relationships between CTV Isolates from Sicily

**Figure 2:**
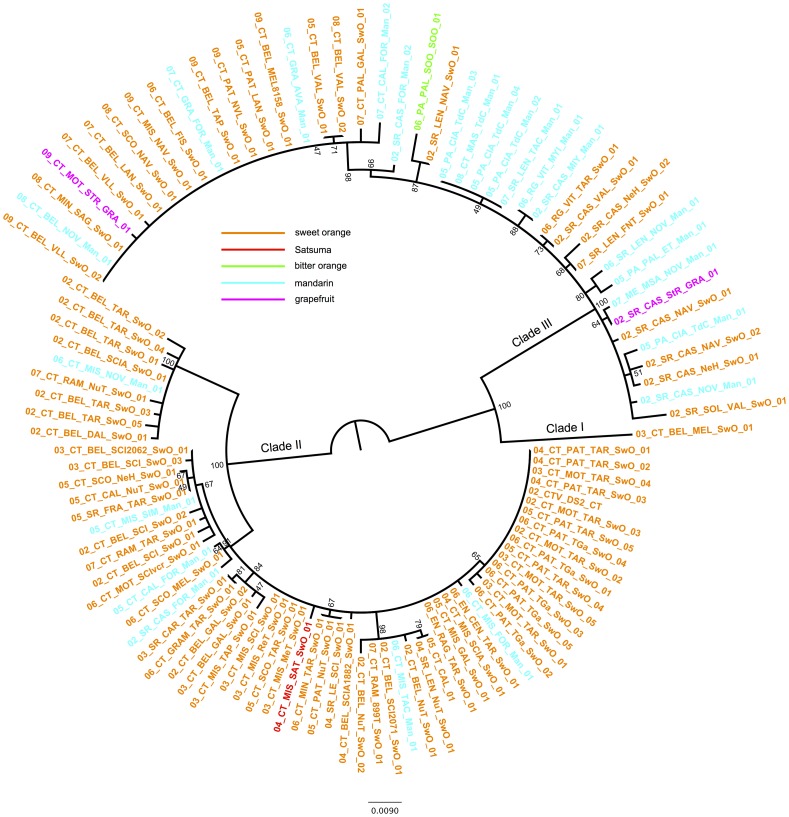
Phylogenetic tree inferred for 108 CTV Sicilian isolates using the p20 gene. The ML tree was constructed using RAxML with the GTR+*Γ*_4_ nucleotide substitution model, introducing three partitions (one for each codon position). Support >40% after 1000 bootstrap replicates is given.

First, the within-isolate CTV population structure was preliminarily estimated by RT-PCR of the p20 gene and single strand conformation polymorphism (SSCP) analysis of 1,789 randomly selected CTV-infected trees (Table S1 in Tables S1). All samples showed simple patterns, composed of two bands corresponding to the two DNA strands (data not shown), which indicated homogeneous within-isolate populations composed of a predominant genetic variant or haplotype [28]. Thus, mixed infections of isolates with divergent haplotypes were not detected among the samples. Next, the consensus nucleotide sequences of the p20 gene of 108 randomly-selected CTV isolates from Sicily were determined and analyzed. No recombination event was detected for this gene, therefore, all sequences were directly used to infer a Maximum Likelihood (ML) phylogenetic tree (Fig. 2). This analysis showed three well supported clades: I comprised only one CTV isolate from Catania, II composed of severe CTV isolates from neighboring provinces (57 isolates from Catania, six from Syracuse and two from Enna) and III which had a wider distribution and included mild CTV isolates from five provinces (20 isolates from Catania, 14 from Syracuse, six from Palermo, one from Messina, and two from Ragusa). The maximum nucleotide distances between isolates were 0.056 and 0.037 within clade II and III, respectively and ranged from 0.083 to 0.114 between isolates from different clades.

### Factors Shaping the Population Genetic Structure of CTV in Sicily

To evaluate how different factors contribute to the genetic variation of CTV, ML trees were constructed based on different hypotheses: H1, the original tree had the same structure as the previously estimated ML tree (Fig. 2); H2, the tree topology is determined by the host species from which isolates were obtained; H3, the tree topology is determined by the geographic origin of isolates; H4, isolates are grouped in the tree according to their sampling date; and H5, virulence (mild vs severe isolates) determines clustering of isolates in the phylogenetic tree. These trees were used to conduct three statistical tests by comparing the polytomic trees H2, H3, H4, and H5 to the reference tree H1 (Table 1). The three tests gave concordant results and showed that the hypothesis H2, H3 and H4 were significantly worse than the null hypothesis H1, whereas H5 was statistically undistinguishable from H1, thus suggesting that the virulence can explain the genetic relationships of the CTV isolates. Indeed, all isolates belonging to clade II were severe whereas isolates of clade III were mild.

**Table 1:** Results of statistical tests of different evolutionary and ecological hypotheses that produce alternative tree topologies for the CTV p20 gene.

Hypotheses	Statistics*		
	SH†Likelihood	K-treedistK-score‡	TOPD/FMTSSplit distance§
H1 (original tree)	−1859.71	0	0
H2 (hosts)	−2394.82*	0.066	0.87
H3 (geographical locations)	−4155.41*	0.067	0.97
H4 (collection years)	−3266.35*	0.066	0.96
H5 (virulence)	−1859.34	0.005	0.70

* These statistical tests compare maximum likelihood trees based on different hypothesis in which the phylogenetic relationships are correlated to the host species (H2), geographical location (H3), collection year (H4) and virulence (H5) with respect to the reference tree (H1).

† Log likelihood for each tree based on the Shimodaira-Hasegawa test. Significant values (*P*<0.01) are indicated by asterisks.

‡ The K-score, the minimum branch length distance from the original tree, was estimated with the program Ktreedist.

§ The split distance, the smallest number of transformations required to obtain one topology from the other, was estimated with the program TOPD/FMTS.

This analysis also revealed that the citrus cultivars did not have a significant influence on the genetic structure of the CTV population neither was this geographically structured (i.e., genetic distances were uncorrelated to the geographic distances). Divergence between CTV isolates was neither correlated to the sampling date. This latter conclusion was confirmed when the clocklikeness of the phylogeny was investigated with the program PATH-O-GEN which gave a very low correlation coefficient between time and tip-to-root distance (0.066), meaning that the number of nucleotide substitution respect to the most recent common ancestor (MRCA) did not increase in a linear manner with time. Nonetheless, the slope of the regression line indicated an average evolution rate of 1.45×10^−4^ substitutions per site and year, a value which is strikingly similar to that estimated from worldwide CTV isolates using a similar Bayesian coalescent approach but covering an interval of 20 years [22].

### Phylogenetic Analysis of Worldwide CTV Isolates Reveals Multiple Introductions of CTV in Sicily

**Figure 3:**
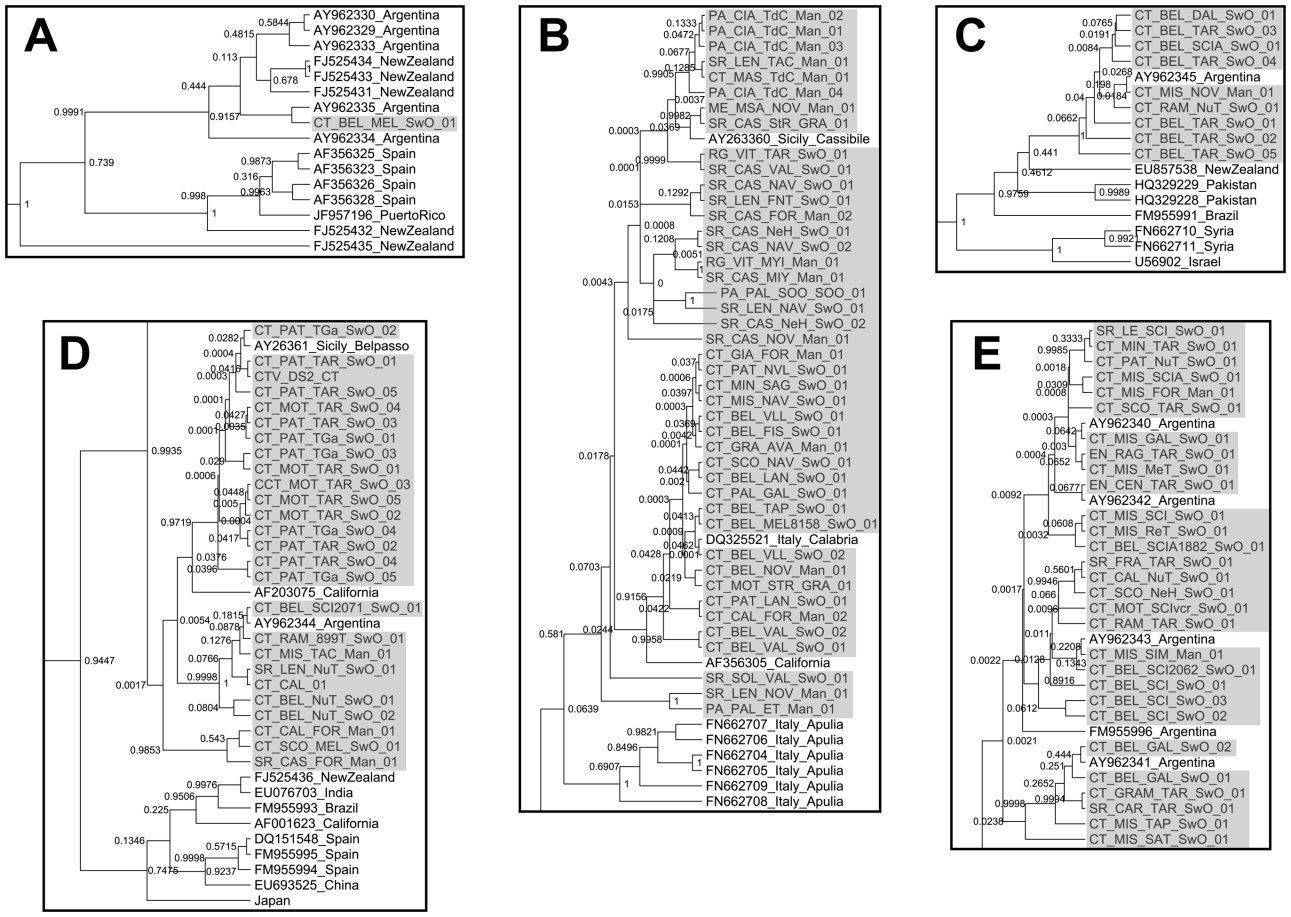
Parts of a Bayesian phylogenetic tree indicating the five clades (A, B, C, D, and E) containing CTV isolates from Sicily. Figure S1 shows all the clades; including also those without the Sicilian isolates. The sequences determined in this work are highlighted in grey background. Node significances are indicated by Bayesian posterior probabilities.

The phylogenetic analysis of 110 CTV Sicilian isolates (108 determined in this work and two from GenBank) and 116 worldwide isolates gave eleven main clades with a high statistical support (Fig. S1). Rather than being monophyletic, as it would be expected from a single introduction event, CTV Sicilian isolates were distributed in five different clades along with isolates from other countries: A, B, C, D and E (Fig. 3) which correlated with the three main clades obtained in Fig. 1 (Clade I corresponded to Clade A, II to C, D and E; and III to B).

Each clade (lineage) is likely to represent a separate introduction of the virus into Sicily, although given the close genetic relationship between CTV isolates within each clade, it cannot be ruled out that some clades might represent multiple introduction events. Clade A had a unique isolate from Sicily and several isolates from Argentina, New Zealand, Spain and Puerto Rico. Clade B contained 44 mild Sicilian isolates which clustered with six isolates from Apulia collected from 2006 to 2008 [29], the region of peninsular Italy where another outbreak occurred in 2002 [19], and one from California. Clade C was composed of nine severe Sicilian isolates which clustered with isolates from Argentina, New Zealand, Pakistan, Brazil, Syria and Israel. Clade D comprised 26 severe Sicilian isolates, one from California and other from Argentina. Finally, Clade E included 30 severe Sicilian and five Argentinean isolates.

Isolates collected early in the outbreaks (2002 and 2003) were from Belpasso, Catania province (clades A, C, D and E), and Cassibile, Syracuse province (clades B and D), which are separated by 80 km in Eastern Sicily. This indicated that all introductions of CTV in Sicily occurred in this region, but it cannot be established whether the virus was introduced independently in both locations in a very short period of time or just in one of them and then it spread out very rapidly to the second location.

Interestingly, the phylogenetic patterns of the Sicilian and the Apulian isolates were clearly different. Thus, within each clade, the Sicilian isolates formed a star-like (unresolved) phylogeny which included also geographically distant CTV isolates with low statistical support for the bifurcating nodes, whereas all isolates from Apulia formed a well-supported and differentiated subclade (within clade B). This latter subclade did not include any isolate from outside Apulia.

The average nucleotide diversity of isolates from the different virus introductions in the island were compared among them and with isolates from the introduction occurred in Apulia, peninsular Italy (Table 2). Nucleotide diversity was very low between isolates from the same introduction in Sicily (<0.010) and in Apulia (0.013). whereas diversity between isolates from different introductions ranged from 0.009 between D and E isolates and 0.127 between C and the Apulian isolates (Table 2).

**Table 2:** Nucleotide diversity of p20 gene of CTV isolates from Italy corresponding to different introductions.

	Sicily A	Sicily B	Sicily C	Sicily D	Sicily E	Apulia
**Sicily A**	*NA* *					
**Sicily B**	0.088±0.017	*0.008±0.001*				
**Sicily C**	0.097±0.017	0.109±0.015	*0.006±0.002*			
**Sicily D**	0.090±0.018	0.088±0.013	0.033±0.008	*0.008±0.002*		
**Sicily E**	0.089±0.017	0.089±0.014	0.032±0.008	0.009±0.002	*0.009±0.002*	
**Apulia**	0.099±0.019	0.020±0.005	0.127±0.018	0.104±0.015	0.104±0.015	*0.013±0.004*

* NA = non-applicable as there is only one CTV isolate.

† Nucleotide diversities and standard errors of CTV isolates proceeding from a possible introduction (in italics) or between CTV isolates from different introductions.

### Dispersion of CTV in Sicily

**Figure 4:**
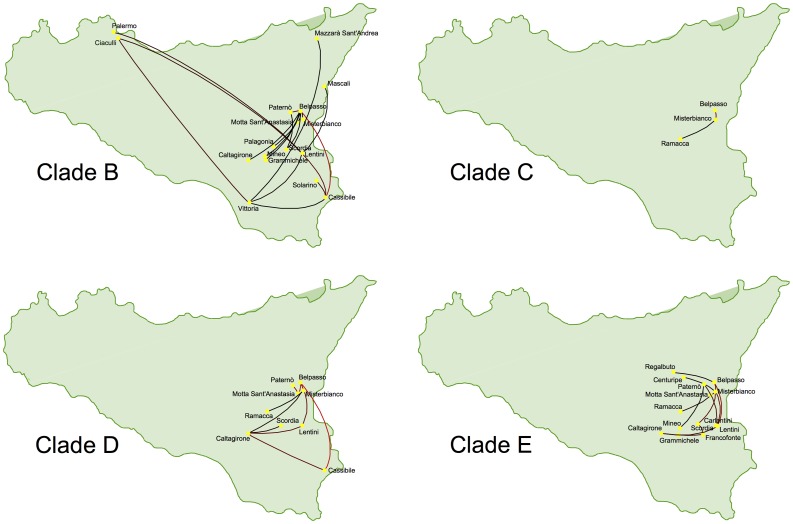
Inferred migration graph for the different introductions of CTV in Sicily associated to clades B, C, D and E reconstructed from the MCC tree. The branches are colored according to the node height values to the colors between the specified maximal (red) and minimal boundary (black). Only introductions associated to clades B, C, D and E are shown since clade A has only a single Sicilian isolate.

The migration patterns of CTV within Sicily Island were estimated from the Bayesian phylogenetic tree and represented in maps (Fig. 4). Each introduction or invasion of CTV deduced from the phylogenetic tree of worldwide CTV isolates (Fig. 3) was considered separately. Clade A had a unique Sicilian CTV isolate from Belpasso indicating that this lineage had a very limited dispersal and was no longer detected. Mild isolates in clade B were first found in several locations of Syracuse province and after a few years spread to neighbouring locations in the Catania province being the only lineage detected after 2007. From 2005 on, this lineage moved to distant locations in the provinces of Palermo (Northwest), where the virus maintained a low prevalence during these years, and in the provinces of Ragusa (South) and Messina (Northeast) but the virus was not detected after 2007 in these provinces. Severe isolates in clade C showed a limited spread of 40 km in the Catania province but they were not found after 2007. Clade D isolates apparently were introduced in Catania and Syracuse occupying an area of ca. 3000 km^2^; but they were not detected after 2007. Finally isolates in clade E also spread from Belpasso in Catania to other locations across the provinces of Catania, Syracuse and Enna, yet restricted to an area of about 2000 km^2^. Also, this lineage was no longer found after 2007.

### Population Genetics of CTV

The three neutrality tests gave negative values, showing a significant deviation from neutrality in the five introductions of CTV in Sicily, except for the Tajima’s *D* test of the clade D introduction (Table 3). This indicates either a decrease of the genetic variation by elimination of deleterious mutations by purifying selection or a rapid population size increase following a bottleneck or founder event. By contrast, the three statistics did not deviate from the neutral evolution expectation for the isolates from continental Italy (Apulia).

**Table 3:** Neutrality tests of p20 gene of CTV isolates from Italy corresponding to different introductions.

Introductions	No. isolates	Fu & Li’s *D*	Fu & Li’s *F*	Tajima’s *D*
**Sicily A**	1	NA*	NA	NA
**Sicily B**	44	−2.850†	−3.033†	−2.001†
**Sicily C**	9	−1.896‡	−2.081‡	−1.745†
**Sicily D**	26	2.341‡	−2.446‡	−1.524
**Sicily E**	30	2.524‡	−2.782†	−2.018†
**Apulia**	7	−0.269	−0.430	−0.771

NA = non-applicable as there is only one CTV isolate.

† P<0.05,

‡ P<0.10,

The strength of the selective constraints for amino acid changes was estimated by computing separately *d_N_* and *d_S_* rates. The values were *d_N_* = 0.022±0.005 and *d_S_* = 0.109±0.020, which translates into a ratio *d_N_*/*d_S_* = 0.202. This value is similar to those obtained for other CTV populations from Spain and California, where CTV has been endemic for long time [21], indicating moderate negative selection for amino acid changes. The statistical estimation of *d_N_* and *d_S_* at each codon site with the FEL method showed that, out of the 154 codons that encode the p20 protein, three adjacent amino acids were under significant positive selection (positions 12, 13 and 14) and 20 were under negative selection (positions 24, 40, 44, 69, 70, 76, 86, 92, 96, 100, 101, 102, 106, 119, 122, 130, 134, 137, 150, and 156). Interestingly, all negatively selected sites are within the p21-like conserved domain of RNA silencing suppressor activity, which corresponds to a computer-predicted alpha-helix [30]. This is a large family of putative suppressors of RNA silencing proteins, P20–P25, from ssRNA positive-stranded viruses in the genera *Closterovirus*, *Potyvirus* and *Cucumovirus*. The three positively selected sites were outside this domain.

Genetic differentiation between CTV populations of Sicily or Italy (including Apulia) and those from other world areas were evaluated by pairwise *F_st_* and the Ks*, Z*, and *S_nn_* tests (Table 4). CTV from Sicily formed a differentiated population with respect to others from Apulia (Italy), Spain, California, New Zealand, Pakistan, and Argentina. Indeed population differentiation between geographically separate CTV populations was the rule, except for those from Spain and California which formed a genetically undifferentiated population. Overall, these results indicate a limited gene flow (migration) between these geographic regions, with the exception of Spain and California.

**Table 4:** *F_st_* values and *Ks* *, *Z* * and *S_nn_* tests between pairs o CTV populations for the p20 gene.

Populations*	*N* †	Argentina	California	N. Zealand	Pakistan	Spain	Apulia
**Argentina**	25						
**California**	20	0.359‡					
**N. Zealand**	7	0.226	0.342				
**Pakistan**	14	0.129	0.537	0.480			
**Spain**	18	0.302	0.0183§	0.216	0.503		
**Italy**	116	0.217	0.155	0.316	0.421	0.123	
**Apulia**	6	0.652	0.258	0.791	0.321	0.650	
**Sicily**	110	0.204	0.182	0.317	0.791	0.321	0.532

* Geographical regions: Argentina, California (USA), New Zealand, Pakistan, Spain, and Italy. Italy has been subdivided between the Sicily island and Apulia (peninsular Italy). The Italian sequences are: 110 from Sicily (108 determined in this work and two from GenBank), six from Apulia (Southeastern of Italian peninsula) and one from Calabria (Southwestern of Italian peninsula).

† *N* = number of CTV isolates.

‡ *F_st_* provides an estimate of the extent of gene flow between populations. A value of zero corresponds to genetically undifferentiate populations, whereas a value of one indicates genetically isolated populations.

§ No significant genetic differentiation (P>0.05) evaluated with the Ks*, Z* and *S_nn_* tests.

## Discussion

We studied the emergence and temporal and spatial evolution of CTV in Sicily with a phylodynamics approach. The Bayesian phylogenetic analysis showed five CTV clades, which included isolates from Sicily and other geographical regions, suggesting that CTV was introduced in Sicily in at least five independent events or several divergent isolates were introduced simultaneously. These introductions occurred in a very short period, probably in 2002, and in two locations, Belpasso and Cassibile (separated 80 km). The geographic origins for these CTV isolates are difficult to track back based on a phylogenetic analysis, due to the lack of a worldwide geographical structure of CTV populations as a result of the international traffic of CTV-infected citrus propagative material [15] and the low evolutionary rate of some CTV genotypes [20]–[22]. Our inquiries revealed that CTV-infected mandarin plants were imported from Spain to Cassibile and that two farmers brought CTV-infected citrus cultivars from California to Belpasso. These events agree with the phylogenetic tree obtained.

The Sicilian CTV isolates within each clade showed an unresolved phylogenetic structure (a star-likestructure with short branches). This is is consistent with a model of recent epidemic, with rapid expansion shortly after virus introduction and minimal selection following a founder event [2], [31]. This interpretation is also consistent with the significant deviations from the neutral evolution model found for the different lineages, which maintained low frequency polymorphism. This result could also result from a very strong negative selection. However, comparison of synonymous and nonsynonymous substitutions suggested a moderate negative selection acting on p20 amino acid sequence similar to that found with CTV isolates from other countries [21]. Twenty (∼13%) of the amino acids were under negative selection whereas only three (∼2%) were under positive selection, which may contribute little to the observed patterns of genetic variation. Thus, while selection seems to affect only a small fraction of the p20 gene the demographic forces derived from genetic drift and from rapid and intense migration are posibly the main factor shaping the CTV population structure. The situation in Sicily was very different to that found in Apulia, the other Italian region analyzed (separated from Sicily ∼450 km, including a three-km sea transect) where CTV was also detected in 2002. CTV isolates from Apulia grouped in a well-differentiated subclade with well resolved nodes and fitting to the neutral evolution model, which suggests a unique introduction in Apulia with a limited migration and most genetic variation being mainly governed by genetic drift after one or several founder events.

No mixed infection with divergent genotypes was detected in spite of (i) the geographic proximity of genetically divergent isolates and the possibility of citrus trees being superinfected by aphid inoculation, and (ii) the lack of known mechanisms for superinfection exclusion between divergent virus strains [32]. A similar analysis in Spain and California showed that mixed infections are rare and probably transient [27], [33]. Co-inoculation of different virulent and avirulent isolates showed that the former usually had higher fitness and became predominant, even if the mild isolate persisted at low frequency [28], [34], [35]. Also, a Spanish CTV isolate containing a predominant mild genotype and a virulent genotype at very low proportion, was found to increase the frequency of the latter after host switch [36]–[38]. Thus it seems plausible that some citrus trees in Sicily experienced different infection events with genetically and biologically divergent isolates, but later one of them became predominant after outcompeting the others.

We found a poor correlation between genetic divergence and time and geographic distance. This could be due to several factors: i) the occurrence of different introductions of genetically similar CTV isolates (as those detected here by phylogenetic analysis) and the predominance of CTV isolates from one of these introductions after 2007; ii) the perennial nature of citrus trees makes it possible that some CTV isolates migrated to other areas and hosts vectored by aphids or humans and, after accumulation of mutations, returned to the original area; and iii) the low evolutionary rate of some CTV genotypes [20]–[22]. In spite of these constraints, phylogeographic analyses provided valuable information on the dispersion patterns following each CTV introduction in Sicily. Except one CTV lineage with only one isolate found, the other four lineages spread out rapidly to neighboring areas in Eastern Sicily, probably vectored by aphids [39]. Although several clades were co-circulating in the same area, only one lineage from a mild CTV isolate persisted after 2007 in Eastern Sicily. This lineage moved with infected buds to distant Northwestern and Southeastern areas of Sicily, but was not detected after 2007 in these areas. After a rapid increase of CTV prevalence, this decreased in the last years, probably because farmers removed symptomatic citrus plants. Interestingly, this case mimics the overall situation in Spain where despite the introduction of virulent isolates, only one lineage corresponding to mild isolates seems to have persisted. The Spanish mild lineage is, nonetheless, distinct from that surviving in Sicily. This contrasts with other geographic regions where virulent isolates are frequent [15] or are increasing in abundance [40].

This is one of the few reports that have used phylogeographical and phylodynamics methods to study the evolution and epidemiology of a plant virus since its emergence. Our study showed the occurrence of multiple introductions of CTV in Sicily followed by a rapid and complex spread pattern with founder effects shaping the CTV population genetic structure. Reconstruction of the migratory routes together with determination of the geographical regions in which the virus become persistent is central to the establishment of effective disease control policies based on surveillance systems.

## Material and Methods

### Virus Isolates

A survey was conducted in all citrus growing areas of the nine provinces of Sicily since the first CTV outbreak in Sicily in 2002 [19], [41] until 2009 (Table S1 in Tables S1). Randomly selected samples of young leaves were collected from 67,922 trees of sweet orange, sour orange, mandarin, and grapefruit cultivars regardless of symptoms. CTV infection was determined by double-antibody-sandwich indirect (DASI) ELISA analysis with the monoclonal antibodies DF1 and 3CA5 (Ingenasa, Madrid, Spain) that recognize all CTV isolates. Each CTV-infected tree was considered as an isolate.

### RNA Purification

Total RNA from young leaves was extracted from 1,789 randomly selected CTV-infected trees (Table S1 in Tables S1). For each sample, approximately 100 mg of leaf tissue was ground in an Eppendorf tube in the presence of 500 µl extraction buffer (200 mM Tris pH 8.5; 1.5% SDS; 300 mM LiCl; 1% sodium deoxycholate; 1% Igepal CA-630; 10 mM EDTA), the mixture was incubated at 65°C for 10 min and then 500 µl of potassium acetate pH 6.5 was added and incubated on ice for 10 min. After a 10-min centrifugation at 13000 rpm, 650 µl of supernatant was transferred into a new tube, mixed with an equal volume of cold isopropanol and incubated for 1 hour at –80°C. After a 10-min centrifugation at 13000 rpm the pellet was washed with 70% ethanol and resuspended in 50 µl of diethylpyrocarbonate-treated distilled water.

### RT-PCR

The p20 gene of CTV isolates was amplified by RT-PCR in one-step reaction in a 25 µl final volume containing 2 µl of total RNAs (template), 20 mM Tris-HCl (pH 8.4), 50 mM KCl, 3 mM MgCl_2_, 0.4 mM dNTPs, 1 µM of primers P20F ad P20R (Vives *et al.* 1999), 4U of RNaseOut, 20 U of SuperScript II reverse transcriptase-RNaseH and 2U of Taq DNA polymerase (Invitrogen, Carlsbad, CA, USA). RT-PCR was under the following conditions: 42°C for 30 min, 94°C for 2 min, 35 cycles of 30 s at 94°C, 30 s at 50°C, and 30 s at 72°C with a final elongation of 4 min at 72°C.

### Genotyping and Biotyping

Within-isolate CTV population structure was assessed by single-strand conformation polymorphism (SSCP) analysis of the RT-PCR products [42]. The consensus nucleotide sequences of the p20 gene of 108 randomly selected CTV isolates were determined from the RT-PCR products in both directions with an ABI PRISM 3100 DNA sequence analyzer (Applied Biosystems). These 108 CTV isolates were biologically characterized by inoculation in sour orange (*Citrus aurantium*) and Mexican lime (*Citrus aurantiifolia*). Based on this, these isolates were classified into two biotypes: i) severe, causing seedling yellows in sour orange and vein corking in Mexican lime, and ii) mild, symptomless in sour orange and a slight vein clearing in Mexican lime (Table S2 in Tables S1).

### Nucleotide Sequence Analysis

Multiple sequence alignment was performed with CLUSTAL W [43]. The nucleotide substitution model which best fits the sequence and nucleotide diversity, assuming that sites have heterogeneous substitution rates described by a gamma distribution with four classes, was inferred with MEGA version 5.05 [44]. Recombination was analyzed with the GARD program available at the Datamonkey Server (www.datamonkey.org) [45] and the RDP3 package [46].

### Population Demography and Selection Analysis

The program DNASP 5.10 [47] was used to estimate Tajima’s *D* [48], Fu & Li’s *D* and *F* [49] statistics to test the mutation neutrality hypothesis [50]. Tajima’s *D* test is based on the differences between the number of segregating sites and the average number of nucleotide differences. Fu & Li’s *D* test is based on the differences between the number of singletons (mutations appearing only once among the sequences) and the total number of mutations. Fu & Li’s *F* test is based on the differences between the number of singletons and the average number of nucleotide differences between every pair of sequences.

DNASP 5.10 was also used to assess genetic differentiation and the gene flow level between Sicily and other geographic regions by using three permutation-based statistical tests: Ks*, Z* and *S_nn_* [51], [52] and the statistic *F_st_* [53].

To study the role of natural selection at the molecular level, the rate of synonymous substitutions per synonymous site (*d_S_*) and the rate of nonsynonymous substitutions per nonsynonymous site (*d_N_*) were analyzed separately. It is assumed that, generally, in a protein, only nonsynonymous changes (producing amino acid changes) are subjected to selection, as they can alter the protein function or structure. The difference between *d_N_* and *d_S_* provides information on the sign and intensity of selection. *d_N_* and *d_S_* were estimated for the whole p20 gene by the Pamilo-Bianchi-Li method [54], implemented in the program MEGA 5.05 [44]. Also, selection across the p20 coding region was studied by estimation of the rates of *d_N_* and *d_S_* at each codon using the Fixed Effects Likelihood (FEL) method [55] available at the Datamonkey Server.

### Phylogenetic Analyses

Maximum Likelihood (ML) phylogenetic analysis was perfomed with the Sicilian CTV sequences using RAxML Pthreads-based version 7.4.2 [58], [59], under the GTR+*Γ*_4_ substitution model introducing three partitions (one for each codon position) and 1000 bootstrap cycles. Based on this ML tree, polytomic trees were constructed representing four different hypotheses: (i) host linked structure, (ii) geography driven structure, (iii) sample date linked structure and (iv) virulence linked structure. The branch lengths of these polytomic trees were optimized and the likelihoods were compared to the best ML tree with a Shimodaira-Hasegawa test [60] implemented in RAxML. Also KTREEDIST [61] was used to calculate the minimun branch lenght distance (or *K* tree score) from one phylogenetic tree to another. Finally, TOPD/FMTS [62] was used to compare the trees regarding their topological congruence using the split distance method. The distance given is the smallest number of transformations required to obtain one topology from the other. PATH-O-GEN version 1.3 (tree.bio.ed.ac.uk/software/pathogen/) was used to investigate the temporal structure of the collected data by using the ML tree as an input together with the sampling dates. PATH-O-GEN performs a linear regression between the genetic distance from the root to the tips and the corresponding collection dates. Temporal structrure was not significant in our data set.

Bayesian phylogenetic analyses were performed with the Sicilian CTV sequences using BEAST v1.6.2 [56] with the GTR+*Γ*_4_ model, introducing three partitions (one for each codon position). The sampling years were introduced and two independent Monte Carlo Markov Chains (MCMCs) were completed with a chain length of 40,000,000 sampling every 1000 trees to establish convergence of all parameters. The BEAST outputs were analyzed using TRACER v1.5 (tree.bio.ed.ac.uk/software/tracer) and the two outputs were combined for increasing the effective sample size (ESS; posterior = 212.1757, likelihood = 997.3781). The sample of the trees was summarized into the maximum clade credibility (MCC) phylogeny using TREEANNOTATOR v1.7.0 (beast.bio.ed.ac.uk/TreeAnnotator), discarding the first 10% of sampled trees as burn-in. This Bayesian tree confirms the structure of the constructed ML tree.

A discrete phylogeographic analysis was done using a continuous-time Markov chain (CTMC) introducing the location attributes and sampling years. The standard phylogeographic model input file for BEAST was modified to set up Bayesian stochastic search variable selection (BSSVS) according to the number of locations. The location states were annotated on an MCC tree using TREEANNOTATOR and visualized using FIGTREE version 1.3.1 (tree.bio.ed.ac.uk/software/figtree/). The location-annotated MCC was converted with SPREAD [57]. The branches are colored according to the node height values with red specified as the maximal and black as the minimal boundary.

The same Bayesian approach was used to construct a phylogenetic tree from sequences of worldwide CTV isolates using BEAST v1.7.4 [56]. Sampling years were not specified, as this information is unknown for many sequences obtained from GenBank. Substitution rates were estimated using the relaxed uncorrelated exponential clock and the strict clock model. For both methods one MCMC was sufficient to obtain an ESS of a good size (relaxed clock: posterior = 640.8366, likelihood = 1099.5541 and strict clock: posterior = 804.5703, likelihood = 1560.6579). The Bayes factor was calculated using TRACER with the likelihood and 1000 bootstrap replicates (*P*(*M_relaxed_*|*D*) = −3903.493±0.288, and *P*(*M_strict_*|*D*) = −3946.271±0.238), and gave a value of *P*(*M_relaxed_*|*D*)/*P*(*M_strict_*|*D*) = 0.989, suggesting the strict clock model to be the best one.

The tree figures in this article were produced with FIGTREE v1.4.0.

The GenBank accession numbers for the *Citrus tristeza virus* sequences reported in this paper are JQ422278 to JQ422385.

## Supporting Information

Figure S1 **Bayesian phylogenetic tree drawn for the p20 gene from 108 CTV isolates from Sicily (sequenced in this work; highlighted in gray) plus 116 worldwide CTV isolates (from GenBank).** Node significances are indicated by Bayesian posterior probabilities. Phylogenetic clades with Sicilian isolates are indicated as A, B, C, D and E.
(TIF)

Tables S1 **This file includes Table S1 and Table S2.**
(DOC)
