# Supplementary figures and images for: Emergence and Phylodynamics of Citrus tristeza virus in Sicily, Italy
Source: PLoS One. 2013 Jun 20;8(6):e66700. doi: 10.1371/journal.pone.0066700 (PMC3688570; doi:10.1371/journal.pone.0066700)

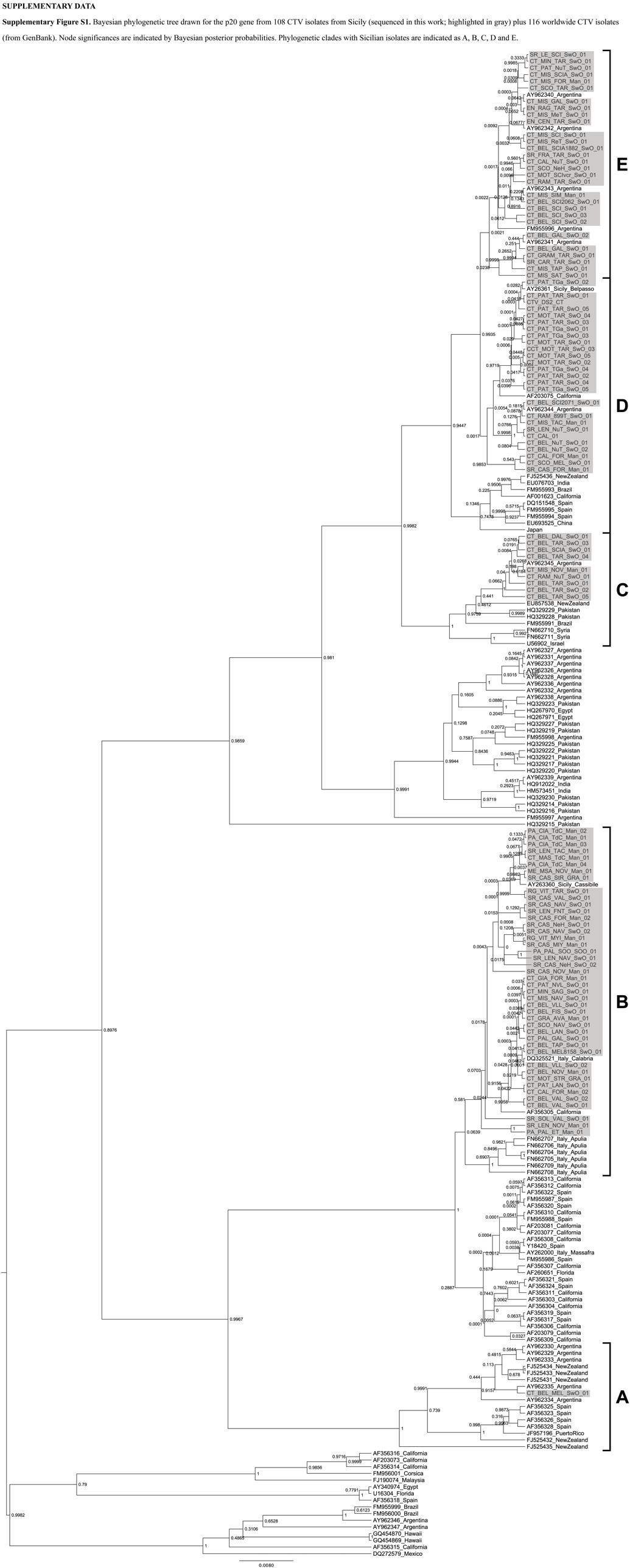

Supplement: Figure S1 — Bayesian phylogenetic tree drawn for the p20 gene from 108 CTV isolates from Sicily (sequenced in this work; highlighted in gray) plus 116 worldwide CTV isolates (from GenBank). Node significances are indicated by Bayesian posterior probabilities. Phylogenetic clades with Sicilian isolates are indicated as A, B, C, D and E. (TIF) [file pone.0066700.s001.tif]
