# Supplementary material for: Emergence and Phylodynamics of Citrus tristeza virus in Sicily, Italy
Source: PLoS One. 2013 Jun 20;8(6):e66700. doi: 10.1371/journal.pone.0066700 (PMC3688570; doi:10.1371/journal.pone.0066700)
Supplement: Tables S1 — This file includes Table S1 and Table S2. (DOC) [file pone.0066700.s002.doc]

**Supplementary Table S1.** Incidence and genotyping of CTV in each Sicilian province for samples collected from 2002 to 2009.

| **Provinces** | **No. tested trees*** | **No. infected trees**† | **No. SSCP**‡ | **No. sequences**§ |
| --- | --- | --- | --- | --- |
| Agrigento | 396 | 0 | 0 | 0 |
| Caltanissetta | 944 | 0 | 0 | 0 |
| Catania | 25,417 | 12, 843 | 752 | 79 |
| Enna | 48 | 11 | 2 | 2 |
| Messina | 319 | 2 | 2 | 1 |
| Palermo | 930 | 102 | 12 | 6 |
| Ragusa | 172 | 27 | 4 | 2 |
| Syracuse | 38,674 | 21,805 | 1,017 | 18 |
| Trapani | 1,022 | 0 | 0 | 0 |
| TOTAL | 67,922 | 34,790 | 1,789 | 108 |

*Number of citrus trees which were analyzed by DASI-ELISA for CTV infection.

†Number of CTV-infected citrus trees.

‡Number of CTV isolates randomly selected for single strand conformation polymorphism (SSCP) analysis.

§Number of CTV isolates randomly selected for sequencing.

**Supplementary Table S2.** CTV isolates whose nucleotide sequences were determined in this work

| **GenBank** | **CTV** | **Citrus** | **Collection** | **Origin in Sicily** | | **CTV** |
| --- | --- | --- | --- | --- | --- | --- |
| **accession** | **Isolate** | **host** | **year** | **Province** | **Area** | **Biotype*** |
| JQ422278 | 06_EN_CEN_TAR_SwO_01 | Sweet orange | 2006 | Enna | Centuripe | Severe |
| JQ422279 | 06_EN_RAG_TAR_SwO_01 | Sweet orange | 2006 | Enna | Regalbuto | Severe |
| JQ422280 | 02_CT_BEL_TAR_SwO_01 | Sweet orange | 2002 | Catania | Belpasso | Severe |
| JQ422281 | 02_CT_BEL_TAR_SwO_02 | Sweet orange | 2002 | Catania | Belpasso | Severe |
| JQ422282 | 02_CT_BEL_TAR_SwO_03 | Sweet orange | 2002 | Catania | Belpasso | Severe |
| JQ422283 | 02_CT_BEL_TAR_SwO_04 | Sweet orange | 2002 | Catania | Belpasso | Severe |
| JQ422284 | 02_CT_BEL_TAR_SwO_05 | Sweet orange | 2002 | Catania | Belpasso | Severe |
| JQ422285 | 02_CT_BEL_NuT_SwO_01 | Sweet orange | 2002 | Catania | Belpasso | Severe |
| JQ422286 | 02_CT_BEL_NuT_SwO_02 | Sweet orange | 2002 | Catania | Belpasso | Severe |
| JQ422287 | 02_CT_BEL_SCI_SwO_01 | Sweet orange | 2002 | Catania | Belpasso | Severe |
| JQ422288 | 02_CT_BEL_SCI_SwO_02 | Sweet orange | 2002 | Catania | Belpasso | Severe |
| JQ422289 | 03_CT_BEL_SCI_SwO_03 | Sweet orange | 2003 | Catania | Belpasso | Severe |
| JQ422290 | 03_CT_BEL_GAL_SwO_01 | Sweet orange | 2003 | Catania | Belpasso | Severe |
| JQ422291 | 02_CT_BEL_GAL_SwO_02 | Sweet orange | 2002 | Catania | Belpasso | Severe |
| JQ422292 | 03_CT_BEL_MEL_SwO_01 | Sweet orange | 2003 | Catania | Belpasso |  |
| JQ422293 | 04_SR_LEN_NuT_SwO_01 | Sweet orange | 2004 | Syracuse | Lentini | Severe |
| JQ422294 | 05_SR_FRA_TAR_SwO_01 | Sweet orange | 2005 | Syracuse | Francofonte | Severe |
| JQ422295 | 02_SR_CAS_FOR_Man_01 | Mandarin | 2002 | Syracuse | Cassibile | Severe |
| JQ422296 | 03_SR_CAR_TAR_SwO_01 | Sweet orange | 2003 | Syracuse | Carlentini | Severe |
| JQ422297 | 04_SR_LE_SCI_SwO_01 | Sweet orange | 2004 | Syracuse | Lentini | Severe |
| JQ422298 | 02_CT_BEL_SCIA_SwO_01 | Sweet orange | 2002 | Catania | Belpasso | Severe |
| JQ422299 | CT_BEL_DAL_SwO_01 | Sweet orange | 2005 | Catania | Belpasso | Severe |
| JQ422300 | 02_CT_BEL_SCI2071_SwO_01 | Sweet orange | 2002 | Catania | Belpasso | Severe |
| JQ422301 | 03_CT_BEL_SCI2062_SwO_01 | Sweet orange | 2003 | Catania | Belpasso | Severe |
| JQ422302 | 04_CT_BEL_SCIA1882_SwO_01 | Sweet orange | 2004 | Catania | Belpasso | Severe |
| JQ422303 | 05_CT_CAL_01 | Sweet orange | 2005 | Catania | Caltagirone | Severe |
| JQ422304 | 05_CT_CAL_NuT_SwO_01 | Sweet orange | 2005 | Catania | Caltagirone | Severe |
| JQ422305 | 05_CT_CAL_FOR_Man_01 | Mandarin | 2005 | Catania | Caltagirone | Severe |
| JQ422306 | 06_CT_GRAM_TAR_SwO_01 | Sweet orange | 2006 | Catania | Grammichele | Severe |
| JQ422307 | 06_CT_MIN_TAR_SwO_01 | Sweet orange | 2006 | Catania | Mineo | Severe |
| JQ422308 | 03_CT_MIS_MeT_SwO_01 | Sweet orange | 2003 | Catania | Misterbianco | Severe |
| JQ422309 | 03_CT_MIS_TAP_SwO_01 | Sweet orange | 2003 | Catania | Misterbianco | Severe |
| JQ422310 | 03_CT_MIS_SCI_SwO_01 | Sweet orange | 2003 | Catania | Misterbianco | Severe |
| JQ422311 | 03_CT_MIS_ReT_SwO_01 | Sweet orange | 2003 | Catania | Misterbianco | Severe |
| JQ422312 | CT_MIS_SCIA_SwO_01 | Sweet orange | 2004 | Catania | Misterbianco | Severe |
| JQ422313 | 04_CT_MIS_SAT_SwO_01 | Sweet orange | 2004 | Catania | Misterbianco | Severe |
| JQ422314 | 05_CT_MIS_GAL_SwO_01 | Sweet orange | 2005 | Catania | Misterbianco | Severe |
| JQ422315 | 06_CT_MIS_NOV_Man_01 | Mandarin | 2006 | Catania | Misterbianco | Severe |
| JQ422316 | 06_CT_MIS_TAC_Man_01 | Mandarin | 2006 | Catania | Misterbianco | Severe |
| JQ422317 | 05_CT_MIS_SIM_Man_01 | Mandarin | 2005 | Catania | Misterbianco | Severe |
| JQ422318 | 06_CT_MIS_FOR_Man_01 | Mandarin | 2006 | Catania | Misterbianco | Severe |
| JQ422319 | CT_MOT_TAR_SwO_01 | Sweet orange | 2007 | Catania | Motta | Severe |
| JQ422320 | 02_CT_MOT_TAR_SwO_02 | Sweet orange | 2002 | Catania | Motta | Severe |
| JQ422321 | 02_CT_MOT_TAR_SwO_03 | Sweet orange | 2002 | Catania | Motta | Severe |
| JQ422322 | 03_CT_MOT_TAR_SwO_04 | Sweet orange | 2003 | Catania | Motta | Severe |
| JQ422323 | 03_CT_MOT_TAR_SwO_05 | Sweet orange | 2003 | Catania | Motta | Severe |
| JQ422324 | 06_CT_MOT_SCIvcr_SwO_01 | Sweet orange | 2006 | Catania | Motta | Severe |
| JQ422325 | 04_CT_PAT_TAR_SwO_01 | Sweet orange | 2004 | Catania | Paternò | Severe |
| JQ422326 | 04_CT_PAT_TAR_SwO_02 | Sweet orange | 2004 | Catania | Paternò | Severe |
| JQ422327 | 04_CT_PAT_TAR_SwO_03 | Sweet orange | 2004 | Catania | Paternò | Severe |
| JQ422328 | 05_CT_PAT_TAR_SwO_04 | Sweet orange | 2005 | Catania | Paternò | Severe |
| JQ422329 | 05_CT_PAT_TAR_SwO_05 | Sweet orange | 2005 | Catania | Paternò | Severe |
| JQ422330 | 05_CT_PAT_NuT_SwO_01 | Sweet orange | 2005 | Catania | Paternò | Severe |
| JQ422331 | 06_CT_PAT_TGa_SwO_01 | Sweet orange | 2006 | Catania | Paternò | Severe |
| JQ422332 | 06_CT_PAT_TGa_SwO_02 | Sweet orange | 2006 | Catania | Paternò | Severe |
| JQ422333 | 06_CT_PAT_TGa_SwO_03 | Sweet orange | 2006 | Catania | Paternò | Severe |
| JQ422334 | 06_CT_PAT_TGa_SwO_04 | Sweet orange | 2006 | Catania | Paternò | Severe |
| JQ422335 | 06_CT_PAT_TGa_SwO_05 | Sweet orange | 2006 | Catania | Paternò | Severe |
| JQ422336 | 07_CT_RAM_NuT_SwO_01 | Sweet orange | 2007 | Catania | Ramacca | Severe |
| JQ422337 | 07_CT_RAM_899T_SwO_01 | Sweet orange | 2007 | Catania | Ramacca | Severe |
| JQ422338 | 07_CT_RAM_TAR_SwO_01 | Sweet orange | 2007 | Catania | Ramacca | Severe |
| JQ422339 | 05_CT_SCO_TAR_SwO_01 | Sweet orange | 2005 | Catania | Scordia | Severe |
| JQ422340 | 05_CT_SCO_NeH_SwO_01 | Sweet orange | 2005 | Catania | Scordia | Severe |
| JQ422341 | 06_CT_SCO_MEL_SwO_01 | Sweet orange | 2006 | Catania | Scordia | Severe |
| JQ422342 | 02_CTV_DS2_CT | Sweet orange | 2002 | Catania | Belpasso | Severe |
| JQ422343 | 06_RG_VIT_TAR_SwO_01 | Sweet orange | 2006 | Ragusa | Vittoria | Mild |
| JQ422344 | 06_RG_VIT_MYI_Man_01 | Mandarin | 2006 | Ragusa | Vittoria | Mild |
| JQ422345 | 07_ME_MSA_NOV_Man_01 | Mandarin | 2007 | Messina | Nova | Mild |
| JQ422346 | 05_PA_CIA_TdC_Man_01 | Mandarin | 2005 | Palermo | Ciaculli | Mild |
| JQ422347 | 05_PA_CIA_TdC_Man_02 | Mandarin | 2005 | Palermo | Ciaculli | Mild |
| JQ422348 | 05_PA_CIA_TdC_Man_03 | Mandarin | 2005 | Palermo | Ciaculli | Mild |
| JQ422349 | 05_PA_CIA_TdC_Man_04 | Mandarin | 2005 | Palermo | Ciaculli | Mild |
| JQ422350 | 05_PA_PAL_ET_Man_01 | Mandarin | 2005 | Palermo | Palermo | Mild |
| JQ422351 | 06_PA_PAL_SOO_SOO_01 | Sour orange | 2006 | Palermo | Palermo | Mild |
| JQ422352 | 02_SR_CAS_FOR_Man_02 | Mandarin | 2002 | Syracuse | Cassibile | Mild |
| JQ422353 | 02_SR_CAS_NOV_Man_01 | Mandarin | 2002 | Syracuse | Cassibile | Mild |
| JQ422354 | 02_SR_CAS_NAV_SwO_01 | Sweet orange | 2002 | Syracuse | Cassibile | Mild |
| JQ422355 | 02_SR_CAS_NAV_SwO_02 | Sweet orange | 2002 | Syracuse | Cassibile | Mild |
| JQ422356 | 02_SR_CAS_NeH_SwO_01 | Sweet orange | 2002 | Syracuse | Cassibile | Mild |
| JQ422357 | 02_SR_CAS_NeH_SwO_02 | Sweet orange | 2002 | Syracuse | Cassibile | Mild |
| JQ422358 | 02_SR_CAS_VAL_SwO_01 | Sweet orange | 2002 | Syracuse | Cassibile | Mild |
| JQ422359 | 02_SR_CAS_MIY_Man_01 | Mandarin | 2002 | Syracuse | Cassibile | Mild |
| JQ422360 | 02_SR_CAS_StR_GRA_01 | Grapefruit | 2002 | Syracuse | Cassibile | Mild |
| JQ422361 | 07_SR_LEN_TAC_Man_01 | Mandarin | 2007 | Syracuse | Lentini | Mild |
| JQ422362 | 06_SR_LEN_NOV_Man_01 | Mandarin | 2006 | Syracuse | Lentini | Mild |
| JQ422363 | 07_SR_LEN_FNT_SwO_01 | Sweet orange | 2007 | Syracuse | Lentini | Mild |
| JQ422364 | 02_SR_LEN_NAV_SwO_01 | Sweet orange | 2002 | Syracuse | Lentini | Mild |
| JQ422365 | 02_SR_SOL_VAL_SwO_01 | Sweet orange | 2002 | Syracuse | Solarina | Mild |
| JQ422366 | 05_CT_BEL_VAL_SwO_01 | Sweet orange | 2005 | Catania | Belpasso | Mild |
| JQ422367 | 08_CT_BEL_VAL_SwO_02 | Sweet orange | 2008 | Catania | Belpasso | Mild |
| JQ422368 | 08_CT_BEL_NOV_Man_01 | Mandarin | 2008 | Catania | Belpasso | Mild |
| JQ422369 | 07_CT_BEL_VLL_SwO_01 | Sweet orange | 2007 | Catania | Belpasso | Mild |
| JQ422370 | 09_CT_BEL_VLL_SwO_02 | Sweet orange | 2009 | Catania | Belpasso | Mild |
| JQ422371 | 07_CT_BEL_LAN_SwO_01 | Sweet orange | 2007 | Catania | Belpasso | Mild |
| JQ422372 | 06_CT_BEL_FIS_SwO_01 | Sweet orange | 2006 | Catania | Belpasso | Mild |
| JQ422373 | 09_CT_BEL_TAP_SwO_01 | Sweet orange | 2009 | Catania | Belpasso | Mild |
| JQ422374 | 09_CT_BEL_MEL8158_SwO_01 | Sweet orange | 2009 | Catania | Belpasso | Mild |
| JQ422375 | 07_CT_CAL_FOR_Man_02 | Mandarin | 2007 | Catania | Caltagirone | Mild |
| JQ422376 | 07_CT_GRA_FOR_Man_01 | Mandarin | 2007 | Catania | Grammichele | Mild |
| JQ422377 | 06_CT_GRA_AVA_Man_01 | Mandarin | 2006 | Catania | Grammichele | Mild |
| JQ422378 | 08_CT_MAS_TdC_Man_01 | Mandarin | 2008 | Catania | Mascali | Mild |
| JQ422379 | 08_CT_MIN_SAG_SwO_01 | Sweet orange | 2008 | Catania | Mineo | Mild |
| JQ422380 | 09_CT_MIS_NAV_SwO_01 | Sweet orange | 2009 | Catania | Misterbianco | Mild |
| JQ422381 | 09_CT_MOT_STR_GRA_01 | Grapefruit | 2009 | Catania | Motta | Mild |
| JQ422382 | 07_CT_PAL_GAL_SwO_01 | Sweet orange | 2007 | Catania | Palagonia | Mild |
| JQ422383 | 05_CT_PAT_LAN_SwO_01 | Sweet orange | 2005 | Catania | Paternò | Mild |
| JQ422384 | 09_CT_PAT_NVL_SwO_01 | Sweet orange | 2009 | Catania | Paternò | Mild |
| JQ422385 | 08_CT_SCO_NAV_SwO_01 | Sweet orange | 2008 | Catania | Scordia | Mild |

*Biotypes: i) severe, causing seedling yellows in sour orange and vein corking in Mexican lime, and ii) mild, symptomless in sour orange and a slight vein clearing in Mexican lime.
